# Difficult and complicated oral ulceration: an expert consensus guideline for diagnosis

**DOI:** 10.1038/s41368-022-00178-0

**Published:** 2022-06-01

**Authors:** Xin Zeng, Xin Jin, Liang Zhong, Gang Zhou, Ming Zhong, Wenmei Wang, Yuan Fan, Qing Liu, Xiangmin Qi, Xiaobing Guan, Zhimin Yan, Xuemin Shen, Yingfang Wu, Lijie Fan, Zhi Wang, Yuan He, Hongxia Dan, Jiantang Yang, Hui Wang, Dongjuan Liu, Hui Feng, Kai Jiao, Qianming Chen

**Affiliations:** 1 State Key Laboratory of Oral Diseases, National Clinical Research Center for Oral Diseases, Chinese Academy of Medical Sciences Research Unit of Oral Carcinogenesis and Management, West China Hospital of Stomatology, Sichuan University, Chengdu, China; 2 College of Stomatology, Chongqing Medical University, Chongqing, 401147 China; 3 Department of Oral Medicine, School and Hospital of Stomatology, Wuhan University, Wuhan, China; 4 Department of Stomatology, Xiang’an Hospital of Xiamen University, School of Medicine, Xiamen University, Xiamen, China; 5 Department of Oral Medicine, Nanjing Stomatological Hospital, Medical School of Nanjing University, Nanjing, China; 6 Department of Oral Medicine, The Affiliated Stomatological Hospital of Nanjing Medical University, Nanjing, China; 7 The Third Affiliated Hospital of Air Force Medical University, Xi’an, China; 8 School and Hospital of Stomatology, Cheeloo College of Medicine, Shandong University, Jinan, China; 9 Beijing Stomatological Hospital, Capital Medical University, Beijing, China; 10 Department of Oral Medicine, Peking University School and Hospital of Stomatology, Beijing, China; 11 Department of Oral Mucosal Diseases, Shanghai Ninth People’s Hospital, College of Stomatology, Shanghai Jiao Tong University School of Medicine, Shanghai, China; 12 Centre of Stomatology, Xiangya Hospital, Cental South University, Changsha, China; 13 Stomatology Hospital, Zhejiang University School of Medicine, Zhejiang Provincial Clinical Research Center for Oral Diseases, Cancer Center of Zhejiang University, Hangzhou, China; 14 Guanghua School of Stomatology, Guangdong Provincial Key Laboratory of Stomatology, Stomatological Hospital, Sun Yat-Sen University, Guangzhou, China; 15 Shanghai Engineering Research Center of Tooth Restoration and Regeneration, Department of Oral Medicine, School of Stomatology, Tongji University, Shanghai, China; 16 Department of Oral Medicine, Hospital of Stomatology, Zunyi Medical University, Zunyi, China; 17 Department of Oral Medicine, School of Stomatology, Capital Medical University, Beijing, China; 18 Department of Emergency and Oral Medicine, School and Hospital of Stomatology, China Medical University, Liaoning Provincial Key Laboratory of Oral Diseases, Shenyang, China; 19 Department of Oral Medicine, Xiangya Stomalogical Hospital, Central South University, Changsha, China; 20 School of Stomatology, The Fourth Military Medical University, Xi’an, Shaanxi China; 21 Stomatology Hospital, School of Stomatology, Zhejiang University School of Medicine, Zhejiang Provincial Clinical Research Center for Oral Diseases, Key Laboratory of Oral Biomedical Research of Zhejiang Province, Cancer Center of Zhejiang University, Hangzhou, China

**Keywords:** Oral diseases, Oral manifestations

## Abstract

The complexity of oral ulcerations poses considerable diagnostic and therapeutic challenges to oral specialists. The expert consensus was conducted to summarize the diagnostic work-up for difficult and complicated oral ulcers, based on factors such as detailed clinical medical history inquiry, histopathological examination, and ulceration-related systemic diseases screening. Not only it can provide a standardized procedure of oral ulceration, but also it can improve the diagnostic efficiency, in order to avoid misdiagnosis and missed diagnosis.

Oral ulceration is quite complicated and diverse. In addition to some cases that can be attributed to local stimulus, including mechanical (sharp edges of residual root or crown, etc.), physical (thermal burns, etc.), or chemical (strong acid or alkali, etc.) factors, most oral ulcers occur due to the combination of both local and systemic causes. The diagnosis and treatment of oral ulcers in various types are tasks of oral specialists. Aside from a large number of common cases, more rare cases of oral ulcers have been recorded. Often, it is difficult to provide immediate and definitive diagnosis for the latter form of cases.

Oral ulceration is characterized by the persistent defect or destruction in the integrity of the oral epithelium, accompanied by variable loss of the underlying connective tissue, resulting in a crateriform appearance.^1^ From the aspects of etiology, causes of oral ulcers are related to traumatic, infectious, allergic factors, and may be associated with skin disease, autoimmune disease, tumor, inflammatory bowel disease, and so on. However, the exact cause is unknown in some cases. For example, recurrent aphthous ulcers (RAU) may be caused by disturbed immune response, genetic predisposition, nutrient deficiency, oral trauma, anxiety or stress. None of these factors has been confirmed.^2^ From the aspects of morphological features, some oral ulcers manifest as typical clinical features. For example, RAU present as well demarcated, oval or round ulcers with a white or yellow pseudomembrane and a surrounding erythematous halo.^3^ Traumatic ulceration is easily diagnosed according to the location and the shape of ulcer corresponding to the stimulating factor. Tuberculosis ulcer is stellate with undermined edges and clear boundary (Fig. 1). But there are many kinds of oral ulcers with no typical clinical features. From the aspects of difficulty degrees, some are easy to diagnose according to the typical clinical manifestations and medical history, such as RAU and traumatic ulceration. Others are difficult to identify due to the insignificant or complicated medical history, and atypical clinical symptoms, comprehensive assessments are needed.

**Fig. 1:**
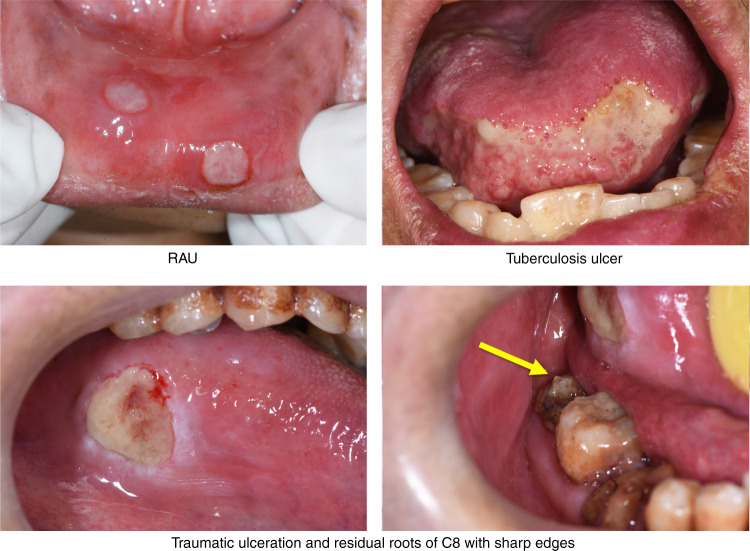
Typical clinical features of some types of oral ulcers

The diversity and complexity of oral ulcerations pose considerable diagnostic challenges to oral specialists. To improve the diagnostic accuracy and timeliness, especially to improve outcomes and survival of those patients with oral ulceration caused by systemic precursor, and to reduce unnecessary financial burden, National Clinical Research Center for Oral Diseases, West China Hospital of Stomatology, Sichuan University takes the lead to summarize the diagnostic work-up for difficult and complicated oral ulcers. The 23 experts in oral medicine from 21 different institutions all over China were invited to discuss and conduct this expert consensus (Fig. 2). This process is decomposed into three parts and will be introduced respectively.

**Fig. 2:**
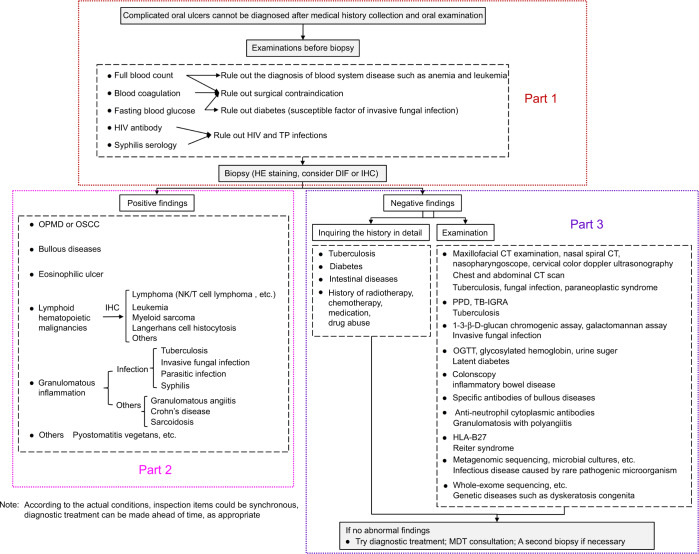
Diagnostic work-up for difficult and complicated oral ulcers

## Part 1

For oral ulceration that cannot be diagnosed after collection of clinical medical history and oral examination, especially those with the course over 2 weeks, or cases which do not respond to 1–2 weeks of treatments, a biopsy should be considered. Note that blood test is necessary before the biopsy, aiming to exclude contraindications. More importantly, blood test can also provide clues of further clinical examination and diagnosis.

**Fig. 3:**
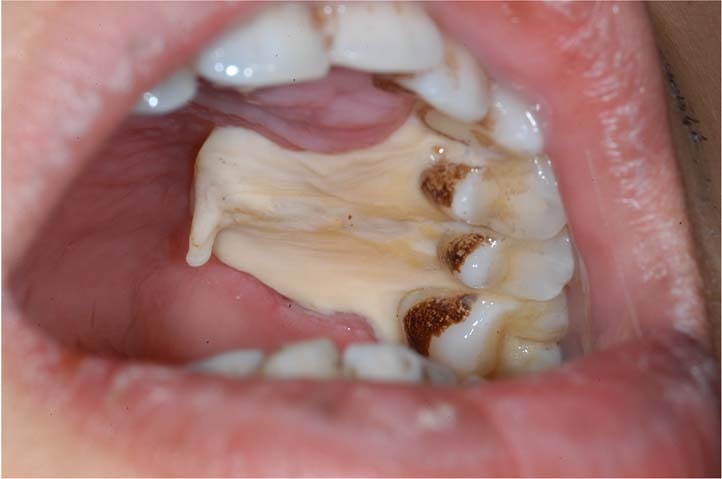
A 22-year-old man with oral ulcers for 3 days. Widespread necrotic ulcers on the left maxillary gingiva, extending to the hard palate, with smooth and thick yellowish-white pseudomembrane. Full blood count showed the percentage of neutrophilic segmented granulocytes decreased significantly (2.0%). Then the bone marrow biopsy and immunotyping revealed acute monocytic leukemia, type M5

Blood tests include full blood count, coagulation, fasting blood glucose level, HIV antibody, and syphilis serology examination. Full blood count can find the trail of blood system diseases. If anemia or leukemia is suspected, the diagnosis should be made by more tests such as blood iron, folate, vitamin B12, bone marrow biopsy, immunotyping, etc (Fig. 3). Blood coagulation and fasting blood glucose are designed to exclude biopsy contraindications. Because hyperglycemia is an important predisposing factor of invasive fungal infection, oral ulcers caused by a fungal infection should be considered in patients with high blood glucose. Detection of HIV antibody and syphilis serology examination help to rule out oral ulceration associated with HIV and syphilis infection. In addition, evaluation for serum specific antibodies, such as Dsg1, Dsg3, BP180, and BP230 before biopsy, is crucial in patients with suspected bullous diseases.

## Part 2

If the diagnostic clues cannot be found by blood test before a biopsy, and there is no contraindication, evaluation of the oral ulceration will enter the Part 2—a biopsy. The following issues need to be considered before and during the biopsy. First, if ulcers involve multiple sites with different morphological characteristics, multiple biopsies should be considered. Second, if bullous diseases are suspected clinically, direct immunofluorescence (DIF) accompanied with HE staining are required. Due to the different submission and specimen handling methods, adequate tissue is needed for both procedures. For HE, the tissue (one from lesional and perilesional site with intact epithelium) in 10% formalin is processed using routine techniques. But DIF specimen is obtained from adjacent normal tissue, Michel’s buffer or normal saline is used as transport medium.^4^ In addition, the specimen requires some normal-appearing tissues and adequate depth, especially the ulcerative lesions clinically suspected to be the lymphoma.

**Fig. 4:**
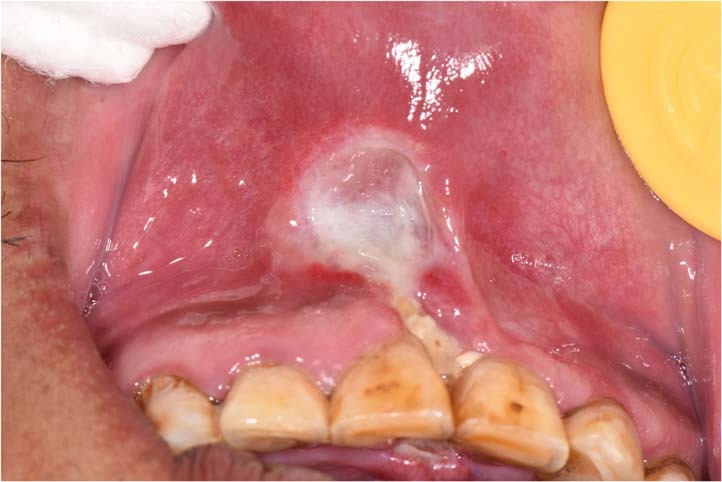
A 25-year-old woman with ulcer for 2 months. Serious erosion and necrosis on the upper labial mucosa, extending to anterior maxillary gingiva covered by yellowish-white pseudomembrane. HE staining and immunohistochemical studies of the biopsy supported the diagnosis of nasal-type extranodal NK/T-cell lymphoma

HE staining of biopsy specimen may suggest the diagnosis in most cases, mainly including cancerous ulcer (carcinoma in situ or oral squamous cell carcinoma), bullous diseases, hematopoietic and lymphoid neoplasm, granulomatous inflammation, acidophilic ulcers, etc. Final diagnosis still need subsequent examinations. If suspected bullous diseases are detected by HE staining, DIF, indirect immunofluorescence, and enzyme-linked immunosorbent assay with recombinant autoantigen should be combined with. If the lesion is suggestive of hematopoietic and lymphoid neoplasm, further examinations such as immunohistochemical assay, T-cell receptors gene rearrangement, bone marrow aspiration, and immunophenotyping are needed (Fig. 4).^5^ Moreover, if granulomatous inflammation is indicated, tuberculosis can be eliminated by acid-fast staining and TB DNA detected by fluorescence quantitative PCR; invasive fungal infection can be excluded by periodic acid-Schiff stain and gomori’s methenamine silver nitrate stain.^6^ Non-infectious granuloma caused by Crohn’s disease and granulomatous vasculitis (Wegener’s granulomatosis) may also be considered. The final diagnosis of Crohn’s disease depends on colonoscopy, gastrointestinal biopsy, and gastrointestinal CT scan.^7^ And diagnosis of granulomatous vasculitis is made by combination of nasal lesion, chest CT, urine routine, and antineutrophil cytoplasmic antibodies.^8,9^

## Part 3

**Fig. 5:**
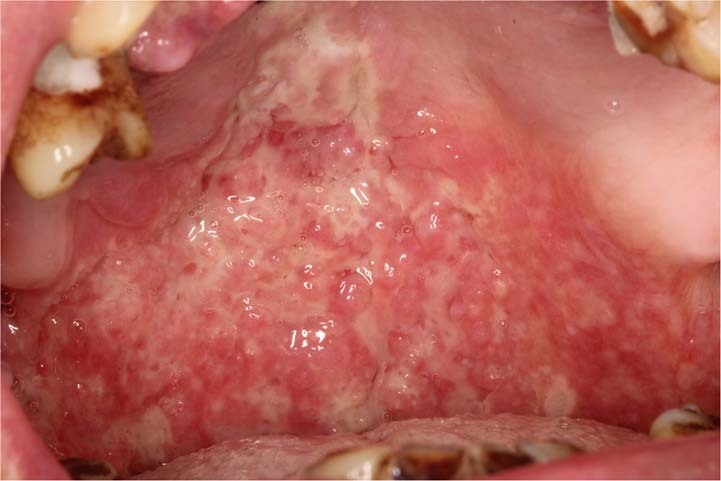
A 72-year-old man with palatal ulceration for 3 months. Widespread ulcers and masses on the palate and maxillary palatal gingiva, extending to the posterior part of the soft palate. He denied the history of systemic diseases. HE staining of repeated oral biopsies showed infiltration of neutrophils and chronic inflammation. Further detailed history inquiry revealed that he developed tuberculosis over a decade ago and was cured after the treatment. We suggested him to take specimen to the superior pathologists for consultation. Granulomatous inflammation containing Langhans-type giant cells was identified, raising the possibility of granulomatous infection. Ziehl-Nielsen staining showed acid-fast bacilli. A chest x-ray revealed bilateral upper lobe consolidation and cavitation, consistent with pulmonary tuberculosis. The definitive diagnosis of oral tuberculosis was established. The ulcers healed, and masses gradually resolved after a combination of isoniazid, rifampicin, pyrazinamide, and ethylaminobutanol for 1 month

Sometimes patients are not eligible to have a biopsy due to contraindications or poor general conditions. Even if the oral biopsy is carried out, a definitive diagnosis is often difficult to obtain. For instance, the pathological findings that “inflammatory ulcer, infiltration of lymphocyte was found in the submucosa” is a common challenge in clinical practice with no specific significance. In this case, we consider taking paraffin-embedded specimens to the superior pathologists for consultation (Fig. 5).

Evaluation of oral ulceration will get into Part 3 if patients with inoperable conditions or the diagnosis remain unestablished after consultation. That is, we step back and perform further screening of ulceration-related systemic diseases.

**Fig. 6:**
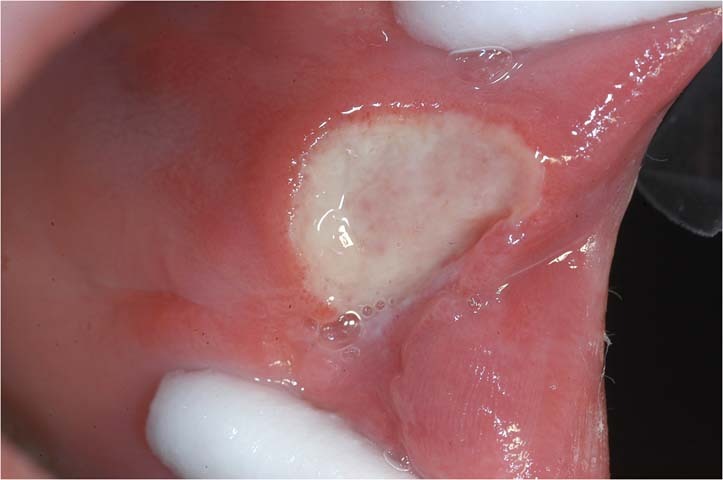
A 13-year-old boy with recurrent oral ulcers for 3 years, reappear 1 month. A 1.3 × 1.5 cm ulcer on the left buccal mucosa, covering with yellowish-white pseudomembrane. Inflammatory ulcer was confirmed pathologically. Further detailed history inquiry of intestinal diseases revealed that he had abdominal pain when oral ulcers occurred. Colonoscopy showed irregular ulcers located on ileocecal and terminal ileum. And he was confirmed as Crohn’s disease by intestinal mucosal biopsy. There was no recurrence of oral ulcer after the treatment of Crohn’s disease

Detailed medical history should be collected again, focusing on common illnesses such as tuberculosis, diabetes, and intestinal diseases (Fig. 6). Moreover, the history of radiotherapy, chemotherapy, medication, drug abuse should also be reconfirmed. Besides, further examinations as appropriate should be considered.

**Fig. 7:**
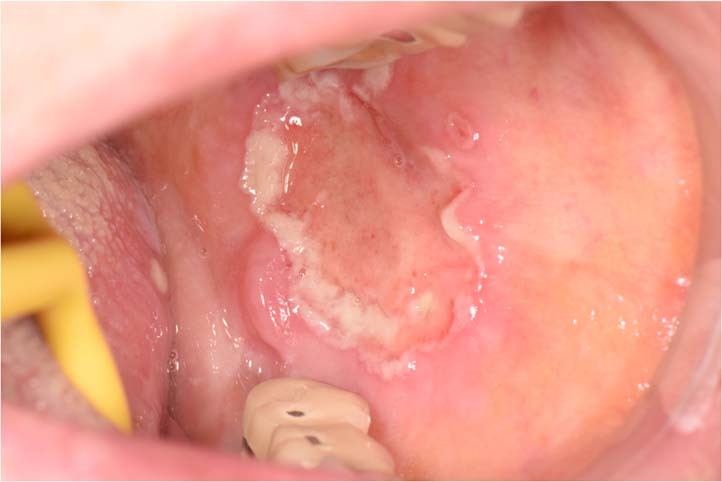
A 72-year-old woman with oral ulcer for 1 month. A 2.0 × 2.4 cm ulcer located on the left buccal mucosa, without apparent inflammation response of surrounding tissues. She was general weakness, and under inoperable conditions, who had the history of angioimmunoblastic T-cell lymphoma, hypertension, diabetes, intracoronary stent implantation and cataract surgery. Due to her high blood glucose level, invasive fungal infection was suspected. The level of 1-3-β-D-glucan and galactomannan increased significantly, probable diagnosis of invasive fungal infection was made. The ulcer healed after infusion of caspofungin

For oral ulcers located on or near the hard tissue, maxillofacial CT examination would find out whether there is bone destruction or not. Otolaryngology consultation, nasal spiral CT, and nasopharyngoscope will be performed if necessary.^5^ Chest CT examination helps to rule out tuberculosis, invasive fungal infection, and paraneoplastic syndrome. Tuberculin skin test (PPD) and interferon gamma release assay (TB-IGRA) are useful to exclude tuberculosis.^10^ Invasive fungal infection can be detected by 1-3-β-D-glucan chromogenic assay and galactomannan (GM) assay. 1-3-β-D-glucan is a cell wall polysaccharide component, which is detected for early diagnosis of fungi, particularly the most common pathogens such as *Aspergillus* and *Candida*, but except for *Zygomycetes* (mainly *Mucor*) and *Cryptococci*. Detection of GM, which is another component of the fungal cell wall, has been used in the diagnosis of invasive aspergillosis.^11^ In addition, serum cryptococcal capsular polysaccharide antigen also has clinical value for the diagnosis of invasive fungal infection (Fig. 7). Oral glucose tolerance test, glycosylated hemoglobin, and urine sugar are the screening methods of diabetes, especially latent or early diabetes. Specific antibodies such as Dsg1, Dsg3, BP180, and BP230 can provide clues to bullous diseases.^12^ Other methods include metagenomic sequencing, microorganism cultivation of ulcerative tissues, immune function measurement, and so on.^13^

If there is no abnormality after the above medical history collection and additional examinations, diagnostic treatment with low-dose and short-term oral glucocorticoids, a second biopsy, or the multi-disciplinary team consultation may be performed if necessary. Screening for genetic diseases by whole-exome sequencing should also be concerned.^14^ Note that inspection items in this diagnosis process may be synchronous according to the actual conditions. Examinations of Part 3 can be made before the biopsy, and a diagnostic treatment could also be performed in advance.

There are numerous causes of oral ulcerations. This diagnosis process only involves common causes without covering all factors. With the development of detection technology and clinical thinking ability, we will further perfect this diagnosis process to provide useful reference and guidance for oral specialists.
